# Coexistence of human pegivirus infection and poor hematopoietic reconstruction in a lymphoma patient after autologous hematopoietic stem cell transplantation: A case report and literature review

**DOI:** 10.1097/MD.0000000000044133

**Published:** 2025-08-22

**Authors:** Jin Kang, Xuan Wang, Jiaofeng Bai, Yuexia Zhang, Bianli Lian, Yali Guo, Ainiwaner Mayire, Yaozhu Pan

**Affiliations:** a Department of Hematology, The 940th Hospital of Joint Logistics Support Force of Chinese People’s Liberation Army, Lanzhou, China; b The First Clinical College of Gansu University of Traditional Chinese Medicine, Lanzhou, China; c Department of Endocrinology, The 940th Hospital of Joint Logistics Support Force of Chinese People’s Liberation Army, Lanzhou, China.

**Keywords:** hematopoietic stem cell transplantation, hepatitis G virus, lymphoma, poor hematopoietic reconstitution

## Abstract

**Rationale:**

Lymphoblastic lymphoma (LBL) is an exceptionally aggressive form of lymphoma. Following the achievement of complete remission through induction therapy, it is imperative to proceed with consolidation and intensive treatment, subsequently followed by hematopoietic stem cell transplantation (HSCT) at the earliest opportunity. Hepatitis G virus (HPgV), an ribonucleic acid virus transmitted via blood, also known as human pegivirus, exhibits a significantly higher positivity rate among HSCT patients compared to the general population. It is widely regarded as nonpathogenic. To date, there have been no documented cases of HPgV-1 infection leading to impaired hematopoietic reconstitution. Nonetheless, the potential for HPgV-1 infection in immunocompromised individuals warrants further case studies for a comprehensive understanding.

**Patient concerns:**

We present a case of B-cell lymphoblastic lymphoma (B-LBL) in an 18-year-old female who presented to our hospital with a 1-month history of right lower limb bone pain.

**Diagnoses:**

B-cell lymphoblastic leukemia/lymphoma (B-LBL).

**Interventions:**

The patient developed HPgV-1 infection accompanied by recurrent fever and poor hematopoietic reconstitution following autologous HSCT.

**Outcomes:**

Within 6 months posttransplantation, the patient’s blood counts remained persistently low: white blood cells fluctuated between 0.77 and 2.65 × 10^9^/L, hemoglobin ranged from 51 to 88 g/L, and platelets varied between 8 and 23 × 10^9^/L. Due to pancytopenia, further consolidation chemotherapy could not be administered. The B-LBL relapsed 8 months posttransplantation, and the patient succumbed to the disease 9 months after transplantation.

**Lessons:**

This case report highlights a rare instance of posttransplant HPgV infection associated with poor hematopoietic recovery in a patient with B-LBL. We surmise that the impaired hematopoietic recovery may be linked to HPgV infection. However, this is a single case report, and further studies are necessary to establish a definitive association.

## 1. Introduction

Lymphoblastic lymphoma (LBL) is a infrequent, extremely aggressive neoplasm of immature B cells that originates from precursor lymphocytes. Patients with unfavorable prognostic features as evaluated by positron emission tomography/computed tomography (PET/CT) and minimal residual disease analysis, should be considered for high-dose chemotherapy and hematopoietic stem cell transplantation (HSCT). Hepatitis G virus (HPgV) is a single-stranded positive-stranded ribonucleic acid virus belonging to the Flaviviridae family, also known as human pegivirus, exhibits a significantly higher positivity rate among HSCT patients compared to the general population, ranging from 18.6% to 41.8%.^[1,2]^ Although HPgV is generally considered nonpathogenic,^[3]^ we encountered a case of B-cell lymphoblastic lymphoma (B-LBL) in our department where the patient developed HPgV-1 infection following autologous hematopoietic stem cell transplantation (auto-HSCT), presenting with recurrent fever and poor hematopoietic reconstitution. Up to date, there have been no reported cases of HPgV-1 infection associated with impaired hematopoietic reconstitution. We surmise that the impaired hematopoietic recovery may be linked to HPgV infection. Nonetheless, the potential for HPgV-1 infection in immunocompromised individuals warrants further case studies for a comprehensive understanding.

## 2. Case presentation

The patient, an 18-year-old female, was admitted to our hospital due to “right lower limb bone pain persisting for 1 month.” On August 8, 2022, the patient experienced unexplained bone pain in the right lower limb. Magnetic resonance imaging of the right knee joint revealed an abnormal signal beneath the right tibial plateau. A biopsy of the upper end of the right tibia indicated B-lymphoblastic leukemia/lymphoma (B-LBL). Bone marrow smear and biopsy showed no tumor cells, a normal chromosomal karyotype, and negative flow cytometry results. PET/CT demonstrated increased metabolic activity in the upper right tibia (SUV6.1). Consequently, she was diagnosed with B-LBL and treated with the hyper-CVAD-A/B regimen (hyperfractionated cyclophosphamide, vincristine, doxorubicin, dexamethasone/methotrexate, and cytarabine) for 4 cycles. Follow-up magnetic resonance imaging of the right knee joint showed improvement in the abnormal signal of the right tibia compared to previous imaging.

On August 24, 2023, the patient underwent auto-HSCT with an infusion of mononuclear cells at 7.88 × 10⁸/kg and CD34 + cells at 4.2 × 10⁶/kg. During the transplantation, the patient experienced moderate gastrointestinal reactions, which improved with symptomatic treatment. Throughout and after the transplantation, she received a total of 10 blood transfusions (6 units of red blood cells and 5.5 units of platelets [PLT]). The absolute neutrophil count (ANC) exceeded 0.5 × 10⁹/L by day 13 posttransplantation, and PLT surpassed 20 × 10⁹/L by day 14. At day 28 posttransplantation, the complete blood count revealed: white blood cells 1.39 × 10⁹/L, ANC 0.91 × 10⁹/L, hemoglobin (HGB) 76 g/L, and PLT 19 × 10⁹/L.

Fever developed 19th day posttransplantation, with temperatures ranging between 37.5°C and 38.5°C. The fever was irregular and accompanied by anorexia and occasional nausea, but no cough, sputum, abdominal pain, or diarrhea. Antibacterial and antifungal treatments were ineffective. PET/CT performed 1 month (+1 m) posttransplantation confirmed metabolic remission. Chest CT showed no evidence of pulmonary infection, and cerebrospinal fluid analysis revealed no abnormalities. Bone marrow smear at + 1 m posttransplantation indicated reduced proliferation of nucleated cells, granulocytes, and megakaryocytes. Flow cytometry of bone marrow cells was negative for malignancy.

**Figure 1.**
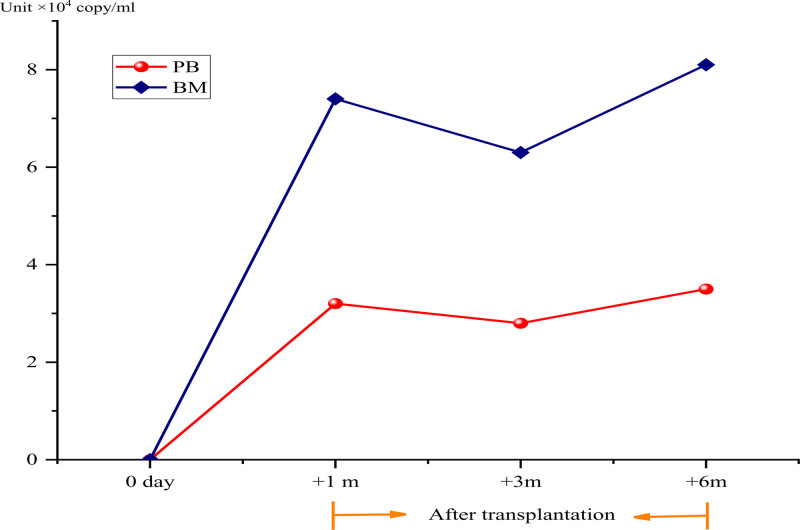
Hepatitis G virus RNA quantification before and after transplantation. RNA = ribonucleic acid.

To identify the cause of the persistent fever, next-generation sequencing (NGS) pathogen testing was performed on blood and bone marrow samples. The results detected only HPgV-1 virus, with no evidence of bacteria, fungi, or other viruses. NGS testing of the pretransplantation autologous stem cell sample showed no pathogens. Quantitative polymerase chain reaction (PCR) for HPgV-1 in blood and bone marrow revealed viral loads of 3.2 × 10⁴ and 7.4 × 10⁴ copies/mL, respectively. Bone marrow reexamination at + 3 and + 6 m posttransplantation showed persistently reduced nucleated cell proliferation, decreased granulocyte and erythroid megakaryocyte proliferation, and negative flow cytometry results. Chromosomal karyotype was normal, and myelodysplastic syndrome-related gene testing was negative. However, PCR detection confirmed persistent HPgV-1 viremia in the bone marrow, and the fever persisted, ranging between 37.5°C and 39.0°C (Fig. 1).

**Figure 2.**
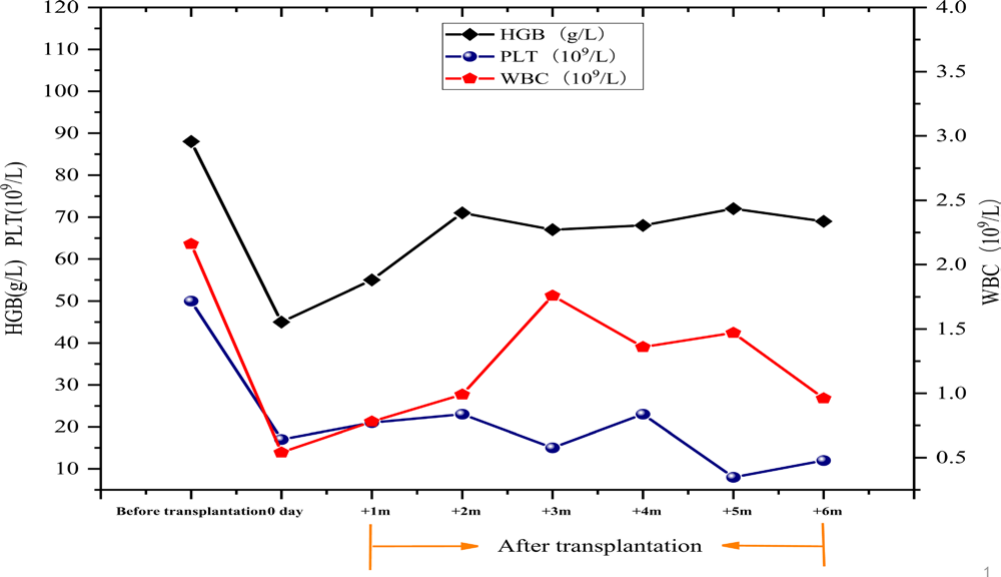
Changes in white blood cells, hemoglobin and platelets in bone marrow and peripheral blood before and after transplantation. HGB = hemoglobin, PLT = platelets, WBC = white blood cells.

Within 6 months posttransplantation, the patient’s blood counts remained consistently low: white blood cells fluctuated between 0.77 and 2.65 × 10⁹/L, HGB ranged from 51 to 88 g/L, and PLT varied between 8 and 23 × 10⁹/L (Fig. 2). Pancytopenia precluded further consolidation chemotherapy, and the B-LBL relapsed 8 months posttransplantation. The patient succumbed to the disease 9 months after transplantation.

## 3. Discussion

B-LBL is a highly aggressive non-Hodgkin lymphoma (NHL) originating from pre-B cells.^[4]^ High-dose chemotherapy combined with auto-HSCT has been extensively applied in the treatment of lymphoma, enabling better treatment outcomes and prolonging patient survival.^[5,6]^ After 28 days of HSCT, if the primary disease is in remission yet the cell counts of 2 or 3 lineages do not meet the engraftment criteria (ANC > 1.5 × 10⁹/L, PLT > 30 × 10⁹/L, and HGB > 85 g/L) and this persists for more than 2 weeks, clinically, poor hematopoietic reconstruction is considered to have occurred.^[7–9]^ The occurrence of poor graft function is associated with factors such as the disease status prior to transplantation, the quantity and quality of hematopoietic stem cells, the bone marrow microenvironment, and viral infections, with an incidence rate of 5% to 27%.^[10]^

In the case of this B-LBL patient, the patient experienced recurrent fever and poor hematopoietic reconstruction posttransplantation. Regarding the fever, infection was initially suspected, and efforts were made to actively search for infection sites and pathogens. However, through corresponding laboratory and imaging examinations, as well as bacterial and fungal cultures of various secretions and excreta, only the pathogenic NGS results indicated the presence of the HPgV-1 virus, while no other bacteria, fungi, or pathogenic microorganisms were detected. Before transplantation, NGS of the autologous stem cell sample did not detect the HPgV-1 virus. We hypothesize that HPgV infection likely occurred during the transplantation process, and the fever may be attributed to HPgV-1 infection.

To further clarify the causes of poor hematopoietic recovery, a bone marrow examination was conducted, revealing reduced proliferation of nucleated cells and granulocytes in the bone marrow, along with a negative minimal residual disease in bone marrow flow cytometry. Both blood and bone marrow HPgV-1 virus PCR showed a high copy number of HPgV. We suspect that the poor bone marrow hematopoiesis may be associated with the HPgV-1 virus.

HPgV was first discovered in the serum of chronic hepatitis patients in the United States in 1995.^[11]^ The virus is widespread in the population, with a nucleic acid positivity rate of approximately 1% to 5% among healthy blood donors in most developed countries, and as high as 20% in some developing countries.^[12]^ HPgV not only replicates in the liver but also demonstrates myelophilic properties. Adam L et al investigated the tissue distribution in animal models infected with HPgV and found that SPgV ribonucleic acid is highly concentrated in only 2 tissues: the spleen and bone marrow. It can infect B cells, T cells, NK cells, monocytes, etc, and is also known as a lymphotropic virus. Thus, the infection rate of HPgV in HSCT patients is high.^[13,14]^ Diem Lan Vu et al^[2]^ analyzed the infection status of HPgV in HSCT patients and found that 32% of patients were infected with HPgV before transplantation, and 41.8% of patients were infected during the follow-up period. The study by Li et al^[15]^ also demonstrated that the infection rate of HPgV in HSCT patients was significantly higher than that in healthy blood donors. Blood transfusion is a high-risk factor for HPgV infection, which can significantly increase the risk of HPgV-1 infection.^[15,16]^ The patient in this case received a total of 10 blood transfusions (red blood cells 6 units and PLT 5.5 units) during and after transplantation, indicating a risk of HPgV infection.

For this case of HPgV infection complicated by poor hematopoietic function recovery after auto HSCT, no relevant reports were found in the literature review. There is still controversy regarding the pathogenicity of HPgV-1. A previous study suggested that HPgV-1 viremia is associated with poor NK cell remodeling after allogeneic HSCT.^[17]^ Some studies have also found that HPgV infection can increase the risk of NHL. The infection rate of HPgV in NHL patients is significantly higher than that in the normal population, and the risk of NHL in HPgV viremia patients is increased by 1.7 times.^[18]^ A meta-analysis involving 1974 NHL patients^[19]^ also showed that HPgV infection is closely related to the NHL disease, indicating that HPgV infection precedes NHL, and the longer the infection duration, the higher the risk factor. It is speculated that HPgV infection reduces lymphocyte activity and proliferation, thereby affecting the body’s immune surveillance and immune response functions, leading to the development of lymphoma. Although HPgV infection is a risk factor for NHL, in a cohort study involving 2948 newly diagnosed lymphoma patients, HPgV infection was not correlated with patient prognosis,^[20]^ meaning that although HPgV infection can increase the risk of NHL, it has no significant impact on patient prognosis.

Studies have reported an association between HPgV infection and aplastic anemia (AA), with the prevalence of HPgV-associated AA estimated at approximately 5.4%. HPgV may indirectly lead to hematopoietic dysfunction of the bone marrow by affecting the homeostasis of the immune system, or it may induce excessive immune responses in the body, causing immune cells to attack hematopoietic stem and progenitor cells in the bone marrow, leading to AA.^[21,22]^ Research by Zhang, Tian et al has demonstrated that following viral infection, CD8 + cytotoxic T cells become overactivated and release cytotoxic factors such as perforin and granzyme B, which induce apoptosis in hematopoietic stem cells. Additionally, viral infection may promote the proliferation of Th1 and Th17 cells while suppressing regulatory T cells, exacerbating the release of inflammatory cytokines (e.g., IFN-γ and TNF-α) and further impairing bone marrow hematopoiesis.^[23]^ However, it has also been suggested that HPgV infection and the development of AA are only incidental associations rather than direct causal relationships.^[24]^ Suggesting that hepatitis viruses may not be a direct factor in the development of the disease, but may in fact exacerbate the immune abnormalities and bone marrow failure of the disease, treatment with a balanced antiviral therapy may be beneficial for hematopoietic restoration, but drugs with potential hematopoietic suppression. Currently, the precise mechanisms by which HPgV induces AA remain unclear and warrant further in-depth investigation.

In conclusion, our case suggests that the fever and poor hematopoietic recovery in the patient after transplantation may be related to HPgV infection. Of course, this is just a case report, and more case studies are needed.

## Acknowledgments

We thank the patient and her family for the publication of this study.

## Author contributions

**Conceptualization:** Jin Kang, Xuan Wang, Jiaofeng Bai, Yaozhu Pan.

**Data curation:** Yuexia Zhang, Bianli Lian.

**Formal analysis:** Yali Guo, Ainiwaner Mayire.

**Funding acquisition:** Yaozhu Pan.

**Supervision:** Yaozhu Pan.

**Validation:** Jin Kang.

**Writing – original draft:** Jin Kang.

**Writing – review & editing:** Xuan Wang, Jiaofeng Bai.
